# America’s Medical Hell**Current Literature*, January, 1911.

**Published:** 1911-04

**Authors:** 


America's Medical Hell.*—One Doctor Norman Barnesby—whom *Current Literature* styles "The Foe of Frenzied Surgery"—like the fabulous "sorry bird that befouls its own nest," according to *C. L.*—"has framed an indictment against the profession as

**Current Literature*, January, 1911.

serious as that drawn by the Flexner report." Let us see about that indictment.

*Imprimis:* Who is Dr. Norman Barnesby? Damfino! Search *me!* I never heard of him.

*Secundus:* It is marvelous that a reputable popular magazine will pay good money for such nauseating Tommy-decomposition as this. It is a crazy-quilt—a thing of shreds and patches, says the *Lancet-Clinic.* It is not a lie "out of the whole cloth," but only in spots, and poorly put together at that.

There are bones in the most wholesome fishes. There are defects in the Bible. There are spots on the sun. No one marvels at that, nor do they condemn the fish because of the bones. Why should this self-sufficient, self-righteous, misfit, single out the bones and serve them to the public, with the sauce of hatred toward the profession he disgraces—intolerance, prejudice, spite and prevarication? Is there a profession anywhere—a society, an organization, church, science, art, politics—in which there are not abuses—errors—that call for reform? Why hadn't this apostle,—"the foe of frenzied surgery,"—the decency to recall some of the thousands of boons, benefactions that medicine and surgery have bestowed upon mankind—the ligature, chloroform, ether, antitoxin, local anesthesia, antisepsis—whereby the average length of life has increased from 33 years, five decades ago, to 48, and keeps his mouth shut? Surgery has been a blessing to mankind. That there are reckless or ignorant men who get into positions through political or money influence and who disgrace their profession is to some extent true; but such instances as this person recites in *Current Literature* should not damn the medical profession, nor be held up to public scorn and made to outweigh the innumerable blessings that have been conferred by the medical profession. The wonder is that the medical profession has accomplished what has been accomplished; has learned what it has learned; has unlocked so many of the secrets of nature and given the world the benefit of this learning and skill in preventing and curing diseases that once were so destructive. That there are too many doctors; too many medical colleges; graduation too easy and license too cheap, goes without saying, and this calls for reform. I really have not the patience to argue with this man—this Judas and Ananias—who thus disgraces himself in such an attempt to besmirch his *alma mater* and his brethren.

---

OVER SIX HUNDRED U. S. troops at Galveston have been subjected to the anti-typhoid inoculation treatment.

